# Penile Metastasis From Castration‐Resistant Prostate Cancer: A Case Report

**DOI:** 10.1002/iju5.70244

**Published:** 2026-08-09

**Authors:** Satoshi Asakura, Yoshihiro Nakagami, Tatsuki Inoue, Yuki Matsui, Kazuhiko Oshinomi, Masakazu Nagata, Takashi Fukagai, Yasuyuki Ohira, Yoshiko Yamochi

**Affiliations:** ^1^ Department of Urology Showa Medical University School of Medicine Tokyo Japan; ^2^ Department of Pathology Showa Medical University School of Medicine Tokyo Japan

**Keywords:** castration‐resistant, neoplasms, neoplasms metastasis, penile neoplasms, prostatic neoplasms

## Abstract

**Introduction:**

Penile metastasis from prostate cancer—generally associated with a poor prognosis—is rare and underreported. We report one relevant case.

**Case Presentation:**

The patient was a 65‐year‐old male with metastatic hormone‐sensitive prostate cancer (prostate‐specific antigen [PSA]: 500 ng/mL) and lung, bone, and lymph node metastases. The patient received luteinizing hormone‐releasing hormone agonist monotherapy. The patient developed castration‐resistant prostate cancer and received androgen receptor signal inhibitor therapy and chemotherapy. As the disease progressed, a mass formed on his penis; biopsy revealed prostate cancer penile metastasis. The patient died 5 months later.

**Conclusion:**

Penile metastasis is rare and typically observed in advanced prostate cancer stages. Awareness may aid in early recognition, guide palliative management, and support patient counseling.

## Keynote Message

Penile metastasis from prostate cancer is extremely rare and has poor prognosis. This case illustrates that prostate cancer can spread to unusual locations even after hormone and chemotherapy treatment. Early recognition of penile masses may help clinicians provide timely palliative care and improve quality of life. Awareness can guide patient counseling and support symptom management.

**Abbreviations**
mCRPC: metastatic castration‐resistant prostate cancer
MRI: magnetic resonance imaging
PSA: prostate‐specific antigen

## 1 Introduction

In 2019, prostate cancer was reported to be the most prevalent cancer affecting males in Japan [1]. Prostate cancer penile metastasis is rare, underreported, and associated with poor prognosis. We report a case of metastatic castration‐resistant prostate cancer (mCRPC) with penile metastasis, and briefly review relevant literature.

## 2 Case Report

A 65‐year‐old man with mCRPC and a history of diabetes mellitus developed penile pain. The patient was referred to our hospital with a prostate‐specific antigen (PSA) level of 500 ng/mL (normal range: < 4.0 ng/mL) at age 61 years. Prostate needle biopsy yielded 12/12 specimens showing acinar adenocarcinoma (Gleason score: 4 + 4). Pathological findings included the proliferation of atypical cells with enlarged nuclei and a pale blue‐colored cytoplasm forming small nests. Fused glandular duct structures were also observed (Figure 1A). Luteinizing hormone‐releasing hormone agonist monotherapy was initiated after a diagnosis of prostate cancer with lung, bone, and lymph node metastases. The initial clinical stage was cT4N1M1c according to the TNM classification. The disease was high‐volume (CHAARTED criteria). Bone scintigraphy revealed multiple metastatic pelvic lesions. PSA nadir was 0.78 ng/mL. Approximately 10 months after hormone therapy initiation, the patient was diagnosed with mCRPC. Despite enzalutamide treatment, PSA levels remained unchanged, and new bone lesions appeared 5 months later. Therefore, docetaxel therapy was initiated. The patient developed febrile neutropenia and lost consciousness after the first course. Granulocyte colony‐stimulating factor preparation was administered during the second docetaxel course, but was discontinued owing to decreased percutaneous oxygen saturation. After 10 courses of cabazitaxel, external beam radiation (78 Gray/39 Fraction) was administered to the prostate and pelvis. The patient participated in a clinical trial, achieving significant PSA level decrease and reduction in abnormal uptake in the pelvis, observed on bone scintigraphy. Ten months after starting the clinical trial, radiographic progression was observed, and the trial was terminated. When the subsequent treatment with abiraterone was initiated, the patient experienced penile pain, a penile rash, and a hard nodule inside the glans. Multiple small nodules were scattered throughout the coronal sulcus (Figure 2). Several tumor markers were evaluated (Table 1). Magnetic resonance imaging (MRI) showed multiple nodules in sagittal penile sections (Figure 3A,B). The glans was replaced by the tumor, although the penile tumor did not show continuity with the prostate on imaging. Transrectal prostate needle biopsy performed before the clinical trial provided a Gleason Score of 4 + 4 for 12/12 specimens. Atypical cells with enlarged nuclei and pale blue acidic cytoplasm proliferated and formed small nests. The penile tumor biopsy specimen was similar to that of the prostate biopsy specimen (Figure 1A,B). No neuroendocrine tumors were detected. Thus, the patient was diagnosed with penile metastasis from prostate cancer. Despite re‐cabazitaxel treatment, the patient died 5 months after penile metastasis identification. Pathological findings at autopsy were similar to those of the initial prostate biopsy. Fused glandular duct structures were observed (Figure 1C). BRCA gene analysis, performed after 10 cabazitaxel cycles, was negative. After external beam radiation therapy, Foundation One Liquid CDx Cancer Genomic Profile was performed, and CDK12 and ROS1 alterations were detected.

**FIGURE 1:**
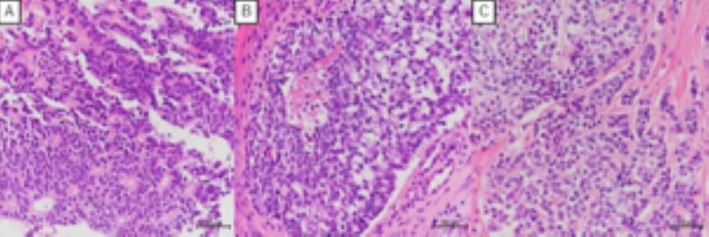
Pathological findings of prostate and penile biopsy. (A) Pathological findings on prostate biopsy include proliferation of atypical cells with enlarged nuclei and small foci of pale blue cytoplasm. Fused glandular duct structures are also seen. (B) Pathological findings on penile biopsy include proliferation of atypical cells with enlarged nuclei and small nests of pale blue cytoplasm. (C) Pathological findings in prostate biopsy at autopsy include proliferation of atypical cells with enlarged nuclei and small foci of pale blue cytoplasm.

**FIGURE 2:**
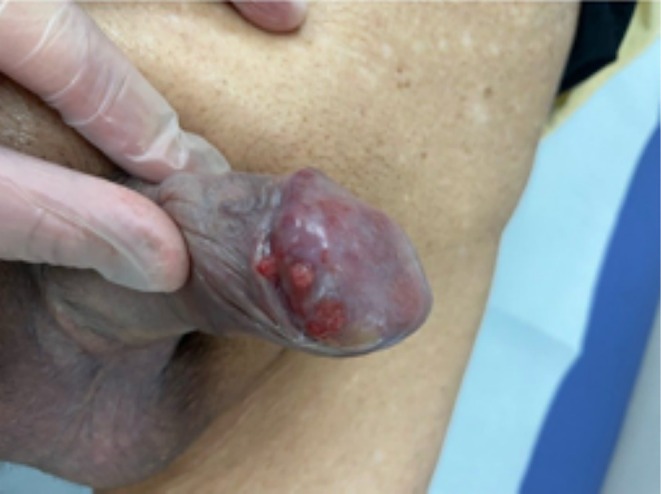
Penile macroscopic findings. Redness and multiple small nodules were observed on the glans.

**TABLE 1:** Patient tumor marker levels.

Tumor marker	Value	Normal range	Interpretation
CEA	8.0 ng/mL	≤ 5.0 ng/mL	Elevated
SCC antigen	0.5 ng/mL	≤ 1.5 ng/mL	Normal
Pro‐GRP	30.7 pg/mL	≤ 81.0 pg/mL	Normal
NSE	29.6 ng/mL	≤ 16.3 ng/mL	Elevated
PSA	466.7 ng/mL	—	Markedly elevated

Abbreviations: CEA, carcinoembryonic antigen; NSE, neuron‐specific enolase; Pro‐GRP, pro‐gastrin‐releasing peptide; PSA, prostate‐specific antigen; SCC, squamous cell carcinoma antigen.

**FIGURE 3:**
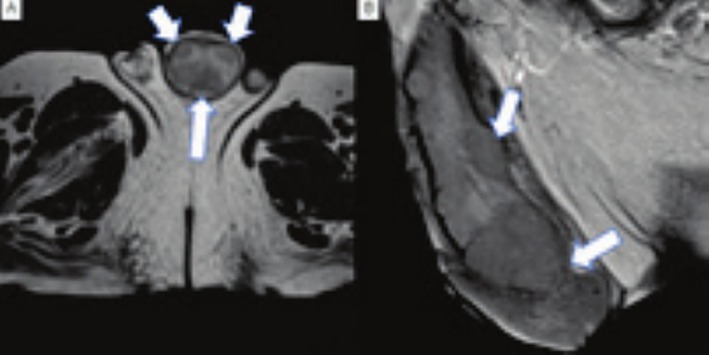
Penile nodules on MRI. (A) Penile plain MRI (T2‐weighted image, horizontal section): Multiple low‐signal nodular shadows observed inside the penis (arrows). (B) Penile plain MRI (T2‐weighted image, sagittal section): Nodular shadows observed inside the penis and low‐signal tumors in the glans (arrows). The nodules are shown using low signal intensity on T2, high signal intensity on diffusion weighting, and low signal intensity on apparent diffusion coefficient.

## 3 Discussion

The first report of penile metastasis from prostate cancer was published in 1972 [2]. Overall, 504 metastatic penile tumor cases have been documented, with most common primary origins being bladder (28.6%), prostate (27.9%), and rectal (12.2%) cancer [3]. Penile metastasis from malignant tumors was first described by Eberth in 1870 [4]. A review in 2006 summarized 372 cases of penile metastasis, including 125 cases (34%) originating from prostate cancer [4]. Between September 2006 and March 2021, 186 additional cases of penile metastasis were reported, of which 72 (38%) originated from prostate cancer [5]. According to previous reports, 137 cases of metastatic penile tumors have been reported in Japan [6]. In 2019, a Japanese report summarized 40 cases of penile metastasis from prostate cancer [7]. The routes of metastasis from prostate to penis include (1) direct invasion, (2) seeding, (3) instrument manipulation, (4) arterial spread, (5) venous retrograde spread, and (6) lymphatic retrograde spread. Retrograde venous spread is considered a common mechanism in which tumor emboli travel from the prostatic venous plexus to the deep dorsal vein of the penis under reversed blood flow conditions caused by increased thoracic or intra‐abdominal pressure [8]. In the present case, no direct continuity between the prostate and penile lesion was observed on imaging; thus, direct invasion was unlikely. Among the proposed mechanisms, retrograde venous spread via the prostatic venous plexus is most plausible. Increased intra‐abdominal pressure may facilitate reversed blood flow, allowing tumor cells to disseminate into the deep dorsal vein of the penis. Hematogenous or lymphatic spread may also contribute to metastasis. However, given the widespread metastatic burden in this patient, a systemic dissemination mechanism was strongly suggested. At the time of penile metastasis diagnosis, the patient had multiple systemic metastases, including bone, lung, and lymph node involvement. No isolated penile lesions were identified, and the disease was considered to be widely disseminated. The poor prognosis associated with penile metastasis is thought to reflect advanced systemic disease burden rather than the penile lesion itself. In the present case, both intrapenile nodules and superficial erythematous lesions of the glans were observed. Therefore, the lesion may represent a combination of penile metastasis and cutaneous involvement rather than cutaneous metastasis alone. The average survival time after diagnosis of penile metastasis from prostate cancer is approximately 6 months [9]; in Japan, only 3 of 25 reported patients survived longer [10]. The present case is notable for several reasons. First, penile metastasis developed in the context of heavily treated metastatic mCRPC after multiple systemic therapy approaches. Second, the case demonstrated a detailed radiologic‐pathologic correlation between the penile lesion and primary prostate tumor. Third, genomic profiling revealed CDK12 and ROS1 alterations, potentially associated with aggressive disease and systemic dissemination. CDK12 loss, particularly, is identified in 3%–7% of metastatic prostate cancers, and CDK12‐deficient prostate cancer has been associated with cancer aggressiveness, genomic instability, and metastatic progression [11]. Although a direct relationship between CDK12 alteration and penile metastasis remains unclear, these findings may partly explain the extensive systemic dissemination observed in our case. In contrast, the clinical significance of ROS1 alteration in prostate cancer remains unclear, although ROS1 rearrangements are recognized oncogenic drivers in several malignancies [12]. Compared with previously reported cases, this case provides several informative features, including extensive treatment history in the mCRPC setting, characteristic MRI findings, pathological concordance between the prostate and penile lesions, autopsy confirmation, and exploratory genomic profiling. These multimodal findings may contribute to a better understanding of the biological behavior and diagnostic features of penile metastasis from advanced prostate cancer.

## 4 Conclusion

Penile metastasis from prostate cancer is rare—typically observed in advanced stages—and has a poor prognosis. This case illustrates that prostate cancer can spread to unusual locations even after hormone and chemotherapy treatment. Although prognosis remains poor, awareness can guide patient counseling and support symptom management.

## Ethics Statement

The protocol for this research project was approved by the Human Research Ethics Committee of Showa Medical University and conforms to the provisions of the Declaration of Helsinki. Approval No. CR2025002.

## Consent

Written informed consent was obtained from the patient for the publication of this article.

## Conflicts of Interest

The authors declare no conflicts of interest.

## Data Availability

Data sharing not applicable to this article as no datasets were generated or analysed during the current study.
